# VetCAST Method for Determination of the Pharmacokinetic-Pharmacodynamic Cut-Off Values of a Long-Acting Formulation of Florfenicol to Support Clinical Breakpoints for Florfenicol Antimicrobial Susceptibility Testing in Cattle

**DOI:** 10.3389/fmicb.2019.01310

**Published:** 2019-06-12

**Authors:** Pierre-Louis Toutain, Pritam Kaur Sidhu, Peter Lees, Ali Rassouli, Ludovic Pelligand

**Affiliations:** ^1^École Nationale Vétérinaire de Toulouse, UMR 1436 Intheres INRA, Toulouse, France; ^2^The Royal Veterinary College, Hawkshead Campus, Hatfield, United Kingdom; ^3^Institute of Computational Comparative Medicine, College of Veterinary Medicine, Kansas State University, Manhattan, KS, United States; ^4^Department of Comparative Biosciences, Faculty of Veterinary Medicine, University of Tehran, Tehran, Iran

**Keywords:** florfenicol, PK/PD cut-off, Antimicrobial Susceptibility Testing, calves, population pharmacokinetics, Monte Carlo simulations

## Abstract

The PK/PD cut-off (PK/PD_CO_) value of florfenicol for calf pathogens was determined for long acting formulations (MSD Nuflor® and a bioequivalent generic product). PK/PD_CO_ is one of the three MICs considered by VetCAST, a sub-committee of the European Committee on Susceptibility Testing (EUCAST), to establish a Clinical Breakpoint for interpreting Antimicrobial Susceptibility Testing (AST). A population model was built by pooling three pharmacokinetic data sets, obtained from 50 richly sampled calves, receiving one of two formulations (the pioneer product and a generic formulation). A virtual population of 5,000 florfenicol disposition curves was generated by Monte Carlo Simulations (MCS) over the 96 h of the assumed duration of action of the formulations. From this population, the maximum predicted MIC, for which 90% of calves can achieve some *a priori* selected critical value for two PK/PD indices, AUC/MIC and T>MIC, was established. Numerical values were established for two bacterial species of the bovine respiratory disease (BRD) complex, *Pasteurella multocida* and *Mannheimia haemolytica*. It was concluded that the PK/PD_CO_ of florfenicol for both AUC/MIC and T>MIC was 1 mg/L.

## Introduction

Florfenicol is an antimicrobial drug (AMD) used extensively to treat Bovine Respiratory Disease (BRD). It's prudent and rational use should be based on the results of Antimicrobial Susceptibility Testing (AST). Clinical breakpoints (CBP) are the MIC values (units mg/L) used by antimicrobial testing laboratories to report qualitatively the results of AST as Susceptible or not. The Veterinary Committee on Antimicrobial Susceptibility Testing (VetCAST) is a recently established sub-committee of the European Union Committee on Susceptibility Testing (EUCAST). EUCAST is the reference committee for AST in human medicine for the EU and VetCAST operates within the guidelines and structure of EUCAST. The VetCAST remit encompasses all aspects of AST of bacterial pathogens of animal origin and animal bacteria with zoonotic potential. In the VetCAST approach (Toutain et al., 2017), CBPs are determined by taking into account at least an epidemiological cut-off (ECOFF) and a PK/PD cut-off (PK/PD_CO_). In addition, a clinical cut-off can also be considered when clinical data are available to link MICs to clinical efficacy (Turnidge and Martinez, 2017). PK/PD_CO_ is defined as the highest possible MIC for which a given percentage of animals in the target population (e.g., 90%) achieve a pre-defined target value, hereafter named PDT (pharmacodynamic target) according to European Medicines Agency (EMA) terminology (European Medicines Agency, 2015). For *Histophilus somni* (HS), *Pasteurella multocida* (PM), and *Mannheimia haemolytica* (MH), possible florfenicol MICs for the wild populations ranged from 0.12 to 2 mg/L; MIC90 values were 0.25 mg/L (HS), 0.5 mg/L (PM), and 1 mg/L (MH) (de Jong et al., 2014).

The Veterinary Antimicrobial Susceptibility Testing (VAST) sub-committee of the Clinical and Laboratory Standards Institute (CLSI), hereafter named CLSI/VAST, historically approved CBP for florfenicol for BRD treatment; selected values were 2, 4, and 8 mg/L, respectively, for Susceptible (S), Intermediate (I), or Resistant (R) (Clinical Laboratory Standards Institute, 2018). To our knowledge, these CBPs were not accompanied by a CLSI/VAST explanatory document to justify the selected values for BRD, although this is now the case for all new CLSI/VAST CBPs. At the time of ascribing these values to florfenicol, CLSI/VAST did not consider PK/PD relationships in the decision taking methods for establishing the CBP. In veterinary medicine, publicly available clinical data on AMD efficacy are generally scarce or non-existent. For florfenicol, several publications have described the clinical efficacy of the formulations (MSD Nuflor® and its generics) considered in the present paper. Results of clinical trials for florfenicol were comprehensively analyzed using a mixed-treatment comparison meta-analysis, which combined evidence from published trials and published estimates of comparative efficacy for 12 AMDs registered for use in the USA (O'Connor et al., 2016). It was concluded that florfenicol was efficacious, ranking fourth of the 12 AMDs investigated. VetCAST, having no access to the company files describing the results of these clinical trials, considers, as do others, that the pivotal information required to establish a CBP is embedded in a PK/PD breakpoint (Turnidge and Paterson, 2007). This is because the PK/PD breakpoint is a hybrid value, incorporating all three principal components (microbiological, pharmacological, and clinical) predicting clinical efficacy. Hence, EUCAST relies on such PK/PD breakpoints to establish CBPs. PK/PD breakpoints should be clearly distinguished from a PK/PD_CO_, in that the latter is derived from PK data only, without any clinical data input. PK/PD_CO_ is established solely by exploring a range of possible (not probable) MICs, and the VetCAST methodology involves computing a series of Probability of Target Attainments (PTA) from plasma concentration-time profiles. This is also the procedure adopted by CLSI/VAST under the name PD_CO_ (Clinical Laboratory Standards Institute, 2018).

VetCAST has re-evaluated the CBP for florfenicol in cattle, in order to provide a proof of concept of its scientific approach, which may differ significantly from that of VAST/CLSI in several respects, including determination of a PK/PD_CO_. Being pivotal for the VetCAST approach, a robust estimation of PK/PD_CO_ requires first the building of a valid population pharmacokinetic (POP PK) model from individual animal data collected from differing sources to quantify typical PK parameters and their between-subject variability (BSV). Simply retrieving, from literature publications, PK parameters estimated by others and aggregating them is not used by VetCAST. Florfenicol and calf pathogens have been selected to illustrate the VetCAST method of meta-analysis (Li et al., 2015). The Non-Linear Mixed Effect Model (NLME) is used to handle unbalanced data (Schoemaker and Cohen, 1996) with one data set having been analyzed using a mono-compartmental model (Sidhu et al., 2014), while more recent data sets have been obtained with a lower limit of quantification (LLOQ) of the analytical technique, thereby providing an extended terminal half-life. This is a very common situation in veterinary medicine, as long-acting (LA) formulations are used extensively (Toutain and Bousquet-Mélou, 2004).

A further aspect of data analysis, specific to VetCAST, is the rationale for selecting the most appropriate PK/PD index, either the time for which plasma concentration remains above the MIC during the dosage interval (*f* T>MIC) or the ratio of Area Under the plasma concentration-time Curve divided by the MIC (*f* AUC/MIC), *f* Cmax/MIC being not considered by EUCAST. The term *f* indicates that these indices should be computed in terms of plasma free drug concentrations. For florfenicol in cattle, the binding to plasma proteins has been reported in several publications, with very disparate results. At the time of model building, the most recent protein binding data for florfenicol were those published by Foster et al. (2016) who concluded “*Florfenicol protein binding was only 5% at the high concentration and was negligible at the low concentrations, representing a fu of essentially 1.0*.” However, others have reported values ranging from 10 to ~25% (Adams et al., 1987; Bretzlaff et al., 1987; Lobell et al., 1994; Sidhu et al., 2014). In light of these data heterogeneity, it was decided to ignore the extent of drug binding in making the present computations, as further explained the Discussion.

Florfenicol is often classified as time-dependent in its killing action and, as for chloramphenicol, T>MIC has been reported as the appropriate PK/PD index (Martinez et al., 2013). However, AUC/MIC has also been proposed as the most appropriate index predictive of clinical efficacy, especially for PM and MH (Sidhu et al., 2014). Actually, it has been shown, using a semi-mechanistic *in silico* model, that AUC/MIC (and not T>MIC) is the most appropriate index, when terminal half-life is relatively long relative to the dosing interval, even for beta-lactam drugs (Nielsen et al., 2011; Kristoffersson et al., 2016). This is the case likewise for florfenicol LA formulations. In the VetCAST project, the best predictive index for florfenicol and its magnitude were investigated from *in silico* simulations using a semi-mechanistic PK/PD model (Nielsen and Friberg, 2013) to replace the classical *in vivo* rodent infection model that, for several decades (Craig, 1998; Andes and Craig, 2002), was used to select the best PK/PD index. VetCAST calculates PDT through an *in silico* dose-fractionation approach (Pelligand et al., 2019).

The aim of the present investigation was to build a population model for florfenicol in cattle, generating by Monte Carlo simulations (MCS) a large number of plasma florfenicol disposition curves (*n* = 5,000). This virtual *in silico* meta-population was used to determine the percentages of animals (PTA) for which a series of possible PDT values would be attainable with differing possible MICs (actually 0.25, 0.5, 1, and 2 mg/L).

## Materials and Methods

Individual calf PK data from three different sources (A = 10, B = 32, C = 8) were used for the POP PK analysis (see Supplementary Material, giving raw data). Source A consisted of 10 calves from a published study (Sidhu et al., 2014). Source B was a drug company (Norbrook Laboratories Limited); it comprised 16 calves enrolled in a cross-over bioequivalence study (MSD Nuflor® and Norbrook Norfenicol® formulations) Norfenicol® being a FDA and EMA approved generic product (Anonymous, 2018a). The 32 data sets were provided by 16 sets for each product, so that for this analysis each of these calves provided two data sets. The third source comprised data from 8 calves in an unpublished study. All calves were in good health and all received a subcutaneous florfenicol dose of 40 mg/kg. Table 1 gives details for the three sources of individual animal data.

**Table 1:** The three data sets considered for florfenicol population pharmacokinetic analysis.

**Data sets**	**Sources**	**Number of calves**	**Total number of plasma samples**	**Number of plasma samples < LLOQ**	**LLOQ mg/L**	**Range of sampling times (h) after dosing**	**Products**
A	Sidhu et al., 2014	10	190	0	0.25	0–80	Nuflor®
B	Company	16	240	12	0.05	0–192	Nuflor®
		16	240	3	0.05	0–192	Generic (Norfenicol®)
C	Unpublished	8	200	7	0.05	0–216	Nuflor®
	Total	50	870	22			

*The 10 calves of the Sidhu' paper were healthy female Aberdeen Angus calves weighing 145–204 kg and aged 79–131 days. The 16 European stock calves from Company were 7 males and 9 females, aged ~5–9 months. They were randomly assigned to two treatment groups with 8 animals in each group, in a manner designed to minimize weight differences. All animals were healthy and physiologically normal. Weights ranged from 136 to 205 kg at selection. The eight unpublished calves were Holstein/Fresian cross-breed and weighed from 108 to 165 kg. Data reported as below the Level of Quantification (< BLQ) obtained after the drug administration was low (2.6%) and were ignored in the present analysis*.

Pharmacokinetic data analyses were carried out using Phoenix® WinNonlin® 8.0 (Pharsight Corporation St. Louis, MO, USA). Data sets obtained from the three sources were analyzed using a NLME model. A two-compartmental model was selected, based on the Likelihood Ratio Test (LRT), the Akaike Information Criterion (AIC), and inspection of different diagnostic plots (vide infra). For the LRT test, the critical value of the χ^2^ distribution considered for a given nominal risk of 0.05, and a given number of degrees of freedom, was obtained using the Excel function CHISQ.INV.RT().

The parameterization of the structural two-compartmental model was of the closed form (Equation 1):

(1) $$\begin{array}{r} \text{C}\left(\text{t}\right)\;=\;\text{A}\;\times \;\text{EXP}\left(-\text{Alpha}\;\times \;\text{t}\right)+\text{B}\;\times \;\text{EXP}\left(-\text{Beta}\;\times \;\text{t}\right)\\ \;-\left(\text{A}+\text{B}\right)\;\times \;\text{EXP}\;\left(-\text{Ka}\;\times \;\text{t}\right) \end{array}$$

where *t* is the time (h) macroconstants, *A* and *B* (μg/ml) are intercepts and *Alpha, Beta*, and *Ka* are rate constants (1/h) associated with the phases of plasma concentration-time profile. Parameterization was in terms of macroconstants and rate constants rather than in terms of clearance and volume of distribution for reasons explained in the Discussion. The five fixed parameters (described as vector Thetas) were estimated and reported as typical values (tv) with coefficient of variation as a measure of precision of the estimate. The random component that describes biological variability around the structural fixed parameters i.e., the BSV across individuals was described by an exponential model of the form (Equation 2):

(2) $$\begin{array}{r} \theta_{1i}\;=\;\theta_{1}\;\times \;\text{Exp}\;\left(\eta_{1i}\right) \end{array}$$

where *θ*_1_ is the typical population value of theta (*A, B, Alpha, Beta*, or *Ka*), *θ*_1*i*_ the value of theta in the ith animal, and η_1*i*_ (eta) the deviation associated with the ith animal from the corresponding theta population value. This exponential model assumes a log-normal distribution of parameters, i.e., that the distribution of the etas is normal in the log-domain, with a mean of 0 and a variance ω^2^ where:

$$\begin{array}{l} \eta \;\approx \;\text{N}\;\left(0,\;\omega^{2}\right) \end{array}$$

Each eta distribution associated to each theta with its own variance ${\;\omega }_{A}^{2}$, $\omega_{Alpha}^{2}\;\omega_{B\;}^{2}$, $\omega_{Beta,\;}^{2}$or $\omega_{Ka}^{2}$was computed, but covariance terms between etas have been ignored (diagonal matrix) to ensure identifiability of the parameters.

The BSV was reported as coefficient of variation in the original scale with the following equation that converts the variance terms (*ω*^2^) to a coefficient of variation (CV%).

(3) $$\begin{array}{r} CV\left(\%\right)=100\times \sqrt{\exp\left(\omega^{2}\right)-1} \end{array}$$

The residual variability was modeled with an additive and a multiplicative component. Like other random-effects, the residual error can be dependent on subject-specific covariate of the analytical technique used to generate plasma concentration (Bonate, 2011). Assuming that the residual mainly reflects variability of the analytical technique, we explored, as a part of the quality control of the merged data sets, what might be the precision of the three analytical techniques used to generate the data i.e., included in the error model was the source of the data as a covariate. It was concluded that differences were not sufficiently large to retain this covariate. Therefore, in the final model, a single residual without covariate was used.

The residual error model without covariate was of the form (Equation 4):

(4) $$\begin{array}{r} Y=\text{f}\left(\theta ,\text{Time}\right)\times \left(1\;+\;\varepsilon_{1}\right)\;+\;\varepsilon_{2} \end{array}$$

with ε1 the multiplicative error term having a mean of 0 and a variance of σ1

$$\begin{array}{l} \varepsilon 1\approx \text{N}\;\left(0,{\sigma 1}^{2}\right) \end{array}$$

and ε2 the common additive error term having a mean of 0 and a variance noted σ2

$$\begin{array}{l} \varepsilon 2\approx \text{N}\;\left(0,{\sigma 2}^{2}\right) \end{array}$$

Sigma1 and Sigma2 were estimated by Phoenix and reported as a CV% for sigma1 and as a STDV for sigma2.

No covariates were included in the final model, as the computed PK/PD_CO_ is expected to cover all sources of biological variability across animals. However, in a preliminary analysis, two covariates were explored, in order to support the merging of the three data sets (*A, B*, and *C)* and the two formulations (Nuflor® and generic). There was no major influence of these covariates (results not shown) and no specific issue linked to the merging of the data sets.

Parameter estimations, with their associated SE and coefficient of variation as a measure of the precision of the estimate, were based on minimizing an objective function value (OFV), using Laplace engine for the Maximum Likelihood Estimation.

As only 22 florfenicol concentrations were reported as BLQ (comprising 2.6% of the whole data set), BLQ data were discounted in the analysis without the risk of introducing bias in the parameter estimates leading to model mis-specification (Byon et al., 2008). For the two-compartment model, when the BLQ incidence was <5%, it was shown that omission of the BLQ data generally did not inflate the bias in the fixed-effect parameters (Xu et al., 2011).

The shrinkage for the etas was estimated by the equation (Karlsson and Savic, 2007):

(5) $$\begin{array}{r} \text{Eta}\;\text{shrinkage}\;=\;1-\frac{\text{SD}\;\left({\text{EBE}}_{\eta }\right)}{\omega } \end{array}$$

where ω is the estimated variability for the population and SD is the SD of the individual values of the Empirical Bayesian Estimates (EBE) of η.

Different diagnostic plots were reviewed to determine whether or not a model was adequate. These included PRED (Population Predicted Value based on population parameter estimates) and IPRED (Individual Predicted value based on individual's ETAs) vs. the DV (Dependent variable) (with and without a log scale) Conditional weighted residuals (CWRES) and individual fitting. The overall adequacy of the 2-compartment PK model was established by plotting the Visual Predictive Check (VPC) i.e., a graphical comparison between the observed data and prediction intervals derived from the simulated data.

Secondary parameters were also computed (terminal half-lives for the first and second phases of drug disposition and contribution of the first and second phases to drug absorption).

Monte Carlo simulations (MCS) of the predicted concentration (IPRED) from the model i.e., simulation of concentration without the error term from 0 to 96 h post administration, with a step of 1 h, were used to generate a meta-population of 5,000 calves. These curves were analyzed using the Non-Compartmental tool of Phoenix to compute the areas under the curve and the time above selected MICs from 0 to 96 h, 96 h being the claimed duration of florfenicol activity after a single SC administration of Nuflor® (Anonymous, 2018b). A PDT of 40% was selected for T>MIC as a default value (Mouton et al., 2012). These metrics were then analyzed with the statistical tool of Phoenix to compute the quantiles of interest (90th) to establish PK/PD_CO_s.

In human medicine, PK/PD indices and their PDT are established primarily in rodent models over a fractionated-dosing interval of 24 h. For florfenicol, such data are not available. Therefore, in this project, an *in silico* approach was used as a surrogate for the dose fractionation trial. Briefly, PD parameters for florfenicol were first estimated by modeling killing curves obtained with *PM* and *MH* with a semi-mechanistic model described by others (Nielsen and Friberg, 2013). Then, the selected PD model was solved with average plasma concentrations predicted by the population model of the present investigation. This *in silico* approach established, retrospectively for the main human AMD classes, all indices derived using the animal model (Nielsen et al., 2011). It was concluded that the best index for florfenicol was AUC/MIC. This component of the project is fully described in a companion paper (Pelligand et al., 2019).

## Results

**Figure 1:**
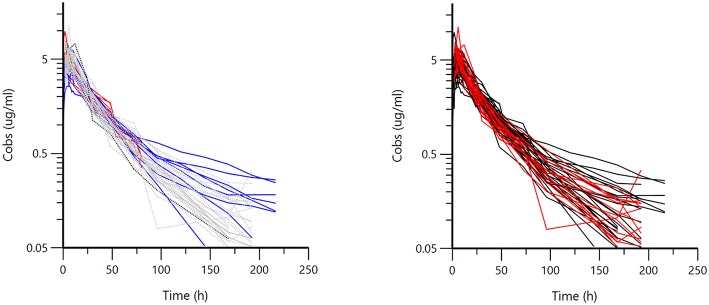
Semi-logarithmic spaghetti plot for 50 calves sorted by sources **(left)** (Red = A, Gray = B, Blue = C) or by formulation **(right)** (Black = Nuflor®, Red = generic). Visual inspection of the plots does not suggest major differences either between the three sources of data or for the two formulations, as seen from the intermingling of the curves.

**Figure 2:**
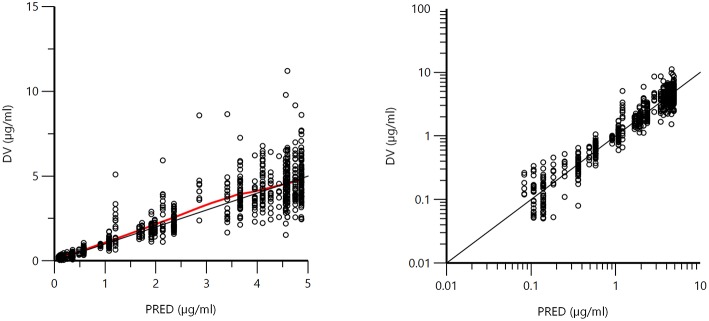
Plot of the dependent variable (DV) i.e., plasma florfenicol concentration (μg/ml) vs. population predicted plasma florfenicol concentrations (PRED) (no random component). The plot illustrates observed vs. fitted values of the model function. Ideally, they should fall close to the line of unity y = x. Arithmetic scale **(left)** and logarithmic scale **(right)**. For both arithmetic and logarithmic scales, data were evenly distributed about the line of identity, indicating no major bias in the population component of the model.

**Figure 3:**
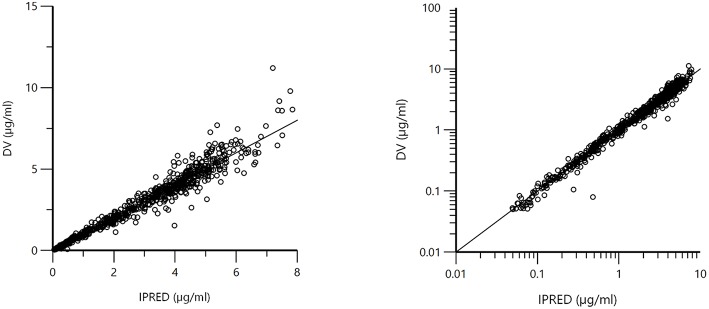
Plot of the dependent variable (DV) i.e., observed plasma florfenicol concentration (μg/ml) vs. individual predicted plasma florfenicol values (IPRED). Individual prediction was obtained by setting random effects to the “*post-hoc*” or empirical Bayesian estimate of the random effects for the individual from which the DV observation was made. Thus, the plots illustrate observed vs. fitted values of the model function. Ideally they should fall close to the line of unity y = x. Arithmetic scale **(left)** and logarithmic scale **(right)**. For both the arithmetic and logarithmic scales, data were evenly distributed about the line of identity, indicating no major bias in the population component of the model.

**Figure 4:**
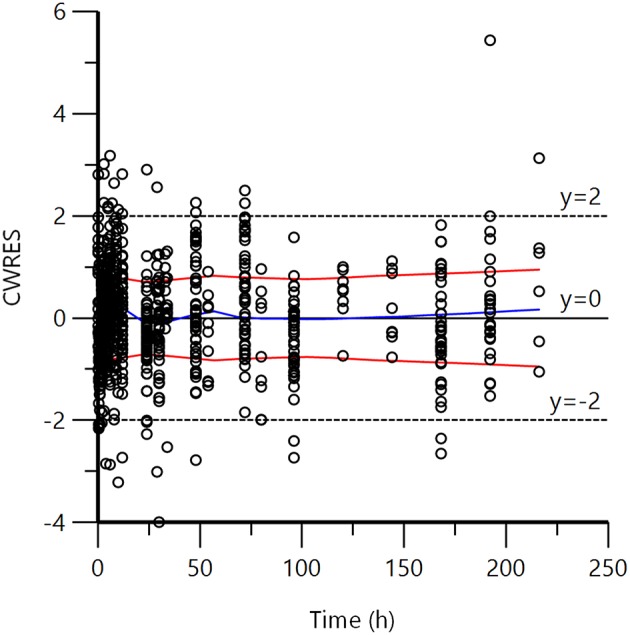
Plot of CWRES (conditional weighted residuals) against time after dose (h). Values of CWRES should be approximately N (0, 1) and hence concentrated between y = −2 and y = +2. Inspection of the figure shows that data were evenly distributed about zero (see the trends as given by the blue line) and the red line (with its negative reflection) did not show any fanning, indicating no bias in the structural model.

**Figure 5:**
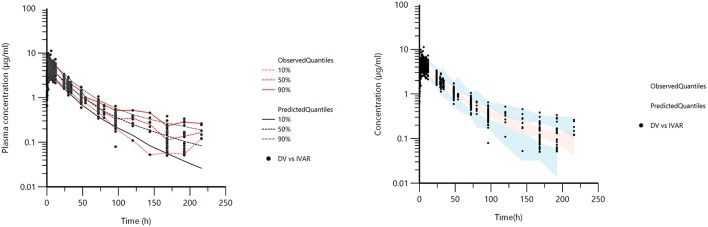
Visual Predictive Check (VPC) obtained with 100 replicates of each animal. The observed quantiles (10, 50, and 90%) were well superimposed on the corresponding predictive check quantiles over the observed data. Theoretically, ~20% of the data should be outside the plotted quantiles. The red lines are 10, 50, and 90% quantiles from the actual observed values. The black lines are the 10, 50, and 90% quantiles from the simulated observations **(left)**. Blue and red shaded **(right)** areas correspond to the 95% confidence interval of the three predicted quantiles.

Figure 1 displays the 50 curves used in the POP PK analysis, sorted either by sources (*n* = 3) or by formulations (*n* = 2). Figures 2–5 are Goodness-of-fit (GOF) plots supporting the 2-comparmental structural model; the exponential model for the random component; and the additive plus multiplicative model for the error sub-model used to analyze the data. To evaluate the adequacy of the developed population model, the VPC plots are presented in Figure 5, which illustrates the 10th, 50th, and 90th percentiles of the simulated distribution compared to the observed values. Typical values of the primary structural parameters of the model (thetas), the secondary parameters (half-life and percentage of the bioavailable dose absorbed during *Alpha* and *Beta* phases), their associated Standard Error (SE) and the SD of the residual for the basic model are presented in Table 2.

**Table 2:** Population primary (Thetas) and secondary parameters and random effects (Omega) for florfenicol in calves obtained with a 2-compartment model.

**THETAS**	**Estimate**	**Units**	**SE**	**CV%**	**2.5% CI**	**97.5% CI**
tvKa	0.975	1/h	0.123	12.66	0.733	1.218
tvA	5.05	μg/ml	0.2368	4.69	4.59	5.52
tvAlpha	0.0442	1/h	0.0041	9.35	0.0361	0.0523
tvB	0.781	μg/ml	0.243	31.13	0.304	1.258
tvBeta	0.0104	1/h	0.0019	18.31	0.0067	0.0141
tvC1MultStdev	0.1397		0.014	10.37	0.111	0.168
tvC1MultStdev	13.970	%				
Covariate analytical method source C	−0.579	Scalar	0.195	−33.73	−0.963	−0.196
Covariate analytical method source B	0.151	Scalar	0.163	107.85	−0.169	0.472
stdev0	0.0152	μg/ml	0.0112	73.64	−0.0068	0.0371
**OMEGA**	**Variance**	**SE**	**BSV (CV%)**	**Shrinkage**		
nKa	0.279	0.076	56.69	0.051		
nAlpha	0.033	0.012	18.23	0.148		
nB	0.036	0.051	19.11	0.544		
nBeta	0.103	0.040	32.97	0.145		
nA	0.080	0.024	28.92	0.050		
**Secondary parameters**	**Estimate**	**Units**	**SE**	**CV%**	**2.5% CI**	**97.5% CI**
Half-life Alpha	15.7	h	1.46	9.35	12.8	18.5
Half-life Beta (t1/2)	66.7	h	12.21	18.31	42.7	90.6
AUC (0-infinity)	183.4	μg^*^h/ml	3.41	1.86	176.1	190.1
Absorption first phase	0.603	Fraction	0.058	9.64	0.489	0.717
Absorption second phase	0.397	Fraction	0.058	14.66	0.283	0.511

*For interpretation of parameters, see Equations (1) (Thetas) and (2) (Omega). AUC was obtained by integrating Equation (1) with estimated tv of thetas parameters. The disposition of florfenicol for the investigated formulations obeys a flip-flop pattern (Discussion) and fraction absorbed during the first vs. the second phase was estimated by computing partial areas associated with the alpha phase (A/Alpha) and the beta phase (B/Beta). Shrinkage was from 0 to 1*.

The coefficient of variation of the multiplicative component of the residual was 14%. The BSV for the estimated parameters was ~20–30% but was 57% for ka, suggesting a homogeneous exposure between animals for these formulations. This is consistent with BSV of AUC, as estimated approximately by others when reporting observed AUC mean and SD (Soback et al., 1995; Sidhu et al., 2014).

Using the developed population pharmacokinetic model and estimated parameters, 5,000 curves were generated by Monte Carlo Simulation (simulated IPREDs taking into account Thetas and Omega but not Sigma, the residual error) over 96 h with a step of 1 h; the simulated dosage regimen 40 mg/kg (single subcutaneous administration). The corresponding AUC from 0 to 96 h and the time for which plasma concentrations remained above selected MICs are given in Table 3.

**Table 3:** AUC (0–96 h), average concentration (μg/ml), and Time (h) above possible MICs ranging from 0.25 to 2 μg/ml for selected quantiles and corresponding value of the T>MIC in % of 96 h, the claimed duration of action of Nuflor®.

	**MIC (μg/ml)**	**Quantiles%**								
		**99**	**95**	**90**	**75**	**50**	**25**	**10**	**5**	**1**
Time above (h) MIC	0.25	74.29	85.54	91.89	95.88	95.92	95.94	95.95	95.96	279.1
Time above (h) MIC	0.5	50.96	58.37	62.46	70.44	80.19	91.1	95.86	95.89	119.88
Time above (h) MIC	1	30.42	35.59	38.7	43.94	50.64	57.72	65.18	70.15	80.21
Time above (h) MIC	2	11.81	16.35	18.9	22.87	28	33.45	39.29	42.63	49.62
Time above MIC (% of 96 h)	0.25	77.38	89.1	95.72	99.87	99.92	99.94	99.95	99.96	290.73
Time above MIC (% of 96 h)	0.5	53.08	60.8	65.06	73.37	83.53	94.9	99.85	99.89	124.87
Time above MIC (% of 96 h)	1	31.69	37.07	**40.31**	45.77	52.75	60.12	67.89	73.07	83.55
Time above MIC (% of 96 h)	2	12.3	17.03	19.68	23.82	29.17	34.85	40.93	44.41	51.69
AUC (0–96 h)	μg^*^h/ml	88.5	103.4	113.1	130.5	153.6	181	211.1	232.5	291
Average concentration (μg/ml) over 96 h	μg/ml	0.92	1.08	**1.18**	1.36	1.6	1.89	2.2	2.42	3.03

*Time above MICs (from 0.25 to 2 μg/ml) was computed from the 5,000 curves generated by MCS using the population model. Bold values indicates values for the selected PK/PD cut-off*.

Data presented in Table 3 indicates that, for a MIC of 1.0 μg/ml, 90% of calves achieved a time above the MIC of at least 38.70 h i.e., a T>MIC of 40.31% of the duration of the assumed florfenicol activity of 96 h. Accepting the claim of the company licensing the pioneer product that the duration of action of Nuflor® is 96 h (Anonymous, 2018b) and a default PDT value of 40% (Mouton et al., 2012), the florfenicol PK/PD_CO_ for T>MIC was 1.0 μg/ml, because, for a higher MIC of 2 μg/ml, a T>MIC of 40% was achieved in only 10% of calves. In accepting AUC/MIC as the appropriate index, the average concentration over 96 h achieved by at least 90% of calves was 1.18 μg/ml. Considering the nearest two-fold MIC value, the PK/PD_CO_ for this index was also 1 μg/ml. This is equivalent to a classical AUC/MIC of 24 h per day in steady-state conditions, as traditionally expressed in human medicine (Toutain et al., 2007). For AUC/MIC values >24 h, the current dosage regimen would not cover 90% of the population; only 10% of calves would be able to achieve an AUC/MIC of 48 h (equivalent to an average plasma concentration of 2 μg/ml over the 96 h interval). An average plasma concentration of 2 μg/ml is equal to the VAST/CLSI CBP.

## Discussion

FDA guidance indicates that population PK modeling (Food Drug Administration, 1999) is the only appropriate tool to allow the meta-analysis of data retrieved from different unbalanced designs i.e., study designs in which all individuals do not supply the same amount of information. For the present analysis, the differences in LLOQ of the analytical technique initially prevented direct comparison of the data set obtained by Sidhu et al. (2014) which fitted a one-compartment model (results not shown) with more recent data obtained with a more sensitive analytical technique and best fitted to a 2-exponential model. Population modeling enabled the older, but nevertheless informative data, to be used to generate a single set of parameters (with SE) for florfenicol. This further enabled generation by MCS of a virtual *in silico* calf population for PK/PD_COs_.

Florfenicol disposition in calves has been investigated following administration by the intravenous route (Varma et al., 1986); PK parameters were estimated with a plasma clearance of 2.85 ml/kg/min, a steady-state volume of distribution, Vss, of 0.75 L/kg, and an elimination t1/2 of 2.86 h. Similar results were reported for different types of cattle, including dairy cattle (Soback et al., 1995), dry cows (Bretzlaff et al., 1987), and steers (Lobell et al., 1994), suggesting no major differences in the florfenicol disposition profile in different classes of cattle. Hence, it is likely that the present findings will be representative of and applicable to differing types of cattle. It is also concluded that a single CBP for cattle can be proposed for these LA formulations. In the present analysis, t1/2 values were much longer than after IV administration, with t1/2 of 16 and 67 h for the *Alpha* and *Beta* phases, respectively. Just as a CBP depends on a specific dosage regimen (Heil and Johnson, 2016), the computed PK/PD_CO_ (in the present analysis) is specific for these LA formulations, administered SC as a single dose of 40 mg/kg with an assumed duration of effect of 4 days. It cannot be assumed that the findings apply to any other dosage regimens and/or other formulations and/or other routes of administration. For example, for another LA florfenicol formulation, it has been shown that the mean differences between a SC and intramuscular (IM) administration were as high as 35 and 63%, respectively, for AUC and Cmax, the IM administration route thus leading to higher florfenicol exposure than subcutaneous dosing (Lacroix et al., 2011). This is typical for veterinary medicine, in which many modalities of AMD administration exist, rendering a universal and robust CBP difficult to propose (Toutain et al., 2017).

The very long terminal t1/2 is explained by flip-flop PK, with the *Alpha* phase corresponding to a first process of relatively slow absorption and the terminal *Beta* phase corresponding to a very slow absorption process. It is concluded that the respective contributions of the *Alpha* and *Beta* phases to the total AUC were ~60% of the bioavailable florfenicol fraction absorbed in the *Alpha* phase and 40% in the *Beta* phase. This second phase is not well-characterized in several publications having a rather high analytical method LLOQ. Nevertheless, the population model allows incorporation of all data in calculating the PK/PD_CO_.

The flip-flop PK profile of the investigated florfenicol formulation is also the basis for choosing to parameterize the model in terms of macroconstants, rather than in terms of clearance and volume of distribution, as is usually the case in population modeling. Indeed, the aim was to simulate 5,000 curves and, whatever the parameterization, the plasma concentration vs. time curves will be the same. From a mechanistic point of view, however, it is important to explore, when estimating PK/PD_CO_, the influence (or not) of the two major covariates involved, namely a possible “formulation” effect (here Nuflor® vs. generic) and a possible “source” effect (here three sources). For both covariates, it is the relative bioavailability that may differ rather than clearance, which is not determinable when only extravascular data are available. To explore the influence of the covariates in question, the *Alpha* and *Beta* slopes are the two parameters to be estimated as primary rather than secondary parameters—hence our parameterization.

The final objective of this population pharmacokinetic analysis was to determine a possible PK/PD_CO_ for florfenicol, this being the pivotal parameter considered by VetCAST in the decision making process for establishing a CBP. PK/PD_CO_ provides insight into the overall PK variability across the targeted populations, because of the relationship between drug exposure and efficacy. This relationship is expressed through PK/PD indices (AUC/MIC ratio or T>MIC) which should achieve critical values to predict clinical efficacy. The magnitude of a PK-PD index providing an appropriate level of predicted response is the PDT (European Medicines Agency, 2015).

As explained in Materials and Methods, no dose-fractionation has been conducted in rodents to determine the best PK/PD index predictive of florfenicol efficacy and in this project an *in silico* approach was used as a surrogate for a dose fractionation trial. It was concluded that the best index was AUC/MIC. This is consistent with the opinion that AUC/MIC is always the most relevant index, when the terminal half-life is long (Nielsen et al., 2011). In addition, it was established that the PDT should be ~24 h per day, indicating that, to achieve an *in silico* bacteriological eradication, the average florfenicol concentration over the 4 days should be equal to the MIC [see Toutain et al. (2007) for explanation of the relationship between PDT expressed in h vs. as a scaling factor]. This is slightly lower than the bactericidal PDT reported from the killing action of florfenicol against MH and PM from modeling of the time-kill data after 24 h exposure of florfenicol to a constant concentration (Illambas et al., 2013).

From the 5,000 curves generated by MCS, the average plasma concentration was estimated to be 1.2 μg/ml (Table 2) and 1 μg/ml is the PK/PD_CO_ value for the AUC/MIC index. An identical PK/PD_CO_ of 1 μg/ml has been derived for florfenicol in pigs, but for a dose of 30 mg/kg (Lei et al., 2018). Florfenicol has been classified as a bacteriostatic drug. It can therefore be argued that T>MIC is also valuable in respect of detection of resistance. However, as quoted by others (Dudley and Ambrose, 2000), the dual aim of achieving a single breakpoint to predict both clinical outcome and avoidance of resistance is likely to fail in many circumstances and constitutes a source of confusion. Nevertheless, the PK/PD_CO_ for T>MIC was computed, assuming that the current dosage regimen should ensure a T>MIC for ~40% of the duration of treatment in 90% of animals: a critical MIC of 1.0 μg/ml was obtained, a value identical with the critical value for the AUC/MIC index. In the present data analysis a PTA of 90% was used to compute the PK/PD_CO_, a quantile that is routinely used for PTA analysis (Turnidge and Paterson, 2007). It should be noted that the quantile 90% is related to the concept of prediction interval and not to the concept of confidence interval. Moreover, the PK/PD_CO_ as applied by VetCAST is not equivalent to the EUCAST PK/PD breakpoint, as the latter takes account additionally of clinical data (Mouton et al., 2012).

In this investigation florfenicol binding to plasma protein was discounted; as discussed in the Introduction, very disparate figures have been reported in cattle, ranging from no binding to binding of 10 to ~25%. Recently, binding was reported with a wide BSV: values in a cohort of 20 calves ranged from 1.88 to 57.5% in 7 day old calves and from 16.8 to 27.8% in 46 day old calves, both at a florfenicol concentration of 1 mg/L (Mzyk et al., 2018). In addressing these differences, we considered that, for the present study, it was appropriate to consider that plasma protein binding of florfenicol was negligible, as suggested by Foster et al. (2016). This approach will render easier any possible future update of computed PK/PD_CO_ for the selected PK/PD index (i.e., AUC/MIC), because the extent of binding is simply a scaling factor for this PK/PD index. Equally important, it seems probable that the variability reported by Mzyk et al. (2018) is not simply associated with some technical issue, but rather actually reflects a true BSV. At present, to the best of our knowledge, this variability is not factored into models used, in veterinary medicine, to compute the PTA using MCS. It is the average value which is adopted. If a wide BSV for protein binding was, in due course, confirmed for florfenicol in cattle, it would be necessary not only to scale our results but to re-run the population model to include this source of variability. To summarize, what has been determined in this paper, as a PK/PD_CO_, is the simplest hypothesis of no plasma protein binding (and thus no variability for this factor) for florfenicol.

In conclusion, any CBP is both dose- and exposure-dependent. In human medicine most AMDs are administered by the oral route and CBPs have a generic value for oral formulations that are relatively similar in terms of the internal exposure they provide. This is unfortunately not the case for veterinary medicine, where CBPs can also be “formulation-dependent.” The formulations, Nuflor® from MSD and its generics, evaluated in this study were all administered by the subcutaneous route, these being the most extensively used formulations and route of administration for florfenicol in cattle. However, other florfenicol formulations and other routes of administration are used in cattle, so that VetCAST CBP is not guaranteed to be applicable to other formulations and/or other routes of administration. These issues are discussed in the VetCAST position paper (Toutain et al., 2017). Finally from a pooled raw data analysis, using a NLME and MCS for florfenicol, a PK/PD_CO_ of 1 mg/L is proposed for the extensively used LA florfenicol formulations investigated.

## Data Availability

The florfenicol raw data are available in the Supplemental File.

## Author Contributions

PL, AR, and PS generated raw data. LP retrieved and validated raw data. P-LT performed the modeling analysis and drafted the paper. All co-authors critically reviewed several drafts of the manuscript.

### Conflict of Interest Statement

The authors declare that the research was conducted in the absence of any commercial or financial relationships that could be construed as a potential conflict of interest.
