# Supplementary material for: VetCAST Method for Determination of the Pharmacokinetic-Pharmacodynamic Cut-Off Values of a Long-Acting Formulation of Florfenicol to Support Clinical Breakpoints for Florfenicol Antimicrobial Susceptibility Testing in Cattle
Source: Front Microbiol. 2019 Jun 12;10:1310. doi: 10.3389/fmicb.2019.01310 (PMC6581757; doi:10.3389/fmicb.2019.01310)
Supplement: Supplementary file 1 [file Data_Sheet_1.pdf]

Individual florfenicol raw data used for the population pharmacokinetic analysis

Time h	Cobs ug/mL	ID	BLQ	MDV	Dose mg/kg
0		1			40
0		1	1	0	
0.25	2.371	1	0	0	
0.5	3.522	1	0	0	
0.75	3.595	1	0	0	
1	3.621	1	0	0	
2	5.476	1	0	0	
3	7.691	1	0	0	
4	6.074	1	0	0	
6	4.548	1	0	0	
8	3.493	1	0	0	
10	3.28	1	0	0	
12	3.149	1	0	0	
24	1.994	1	0	0	
29	1.852	1	0	0	
33	1.55	1	0	0	
48	1.18	1	0	0	
54	0.99	1	0	0	
72	0.807	1	0	0	
80	0.417	1	0	0	
0		2			40
0		2	1	0	
0.25	2.394	2	0	0	
0.5	3.241	2	0	0	
0.75	3.601	2	0	0	
1	3.923	2	0	0	
2	4.925	2	0	0	
3	5.422	2	0	0	
4	5.158	2	0	0	
6	4.829	2	0	0	
8	3.955	2	0	0	
10	3.713	2	0	0	
12	3.488	2	0	0	
24	1.975	2	0	0	
29	1.677	2	0	0	
33	1.532	2	0	0	
48	1.269	2	0	0	
54	0.922	2	0	0	
72	0.803	2	0	0	
80	0.467	2	0	0	
0		3			40
0		3	1	0	
0.25	2.745	3	0	0	
0.5	3.937	3	0	0	
0.75	4.1	3	0	0	
1	4.133	3	0	0	
2	4.844	3	0	0	
3	5.025	3	0	0	
4	4.916	3	0	0	
6	4.447	3	0	0	
8	3.495	3	0	0	

10	3.32	3	0	0	
12	3.048	3	0	0	
24	2.126	3	0	0	
29	1.73	3	0	0	
33	1.455	3	0	0	
48	1.219	3	0	0	
54	0.864	3	0	0	
72	0.767	3	0	0	
80	0.462	3	0	0	
0		4			40
0		4	1	0	
0.25	3.043	4	0	0	
0.5	3.027	4	0	0	
0.75	4.733	4	0	0	
1	4.93	4	0	0	
2	5.274	4	0	0	
3	5.634	4	0	0	
4	4.987	4	0	0	
6	4.682	4	0	0	
8	4.553	4	0	0	
10	4.359	4	0	0	
12	4.025	4	0	0	
24	2.582	4	0	0	
29	2.433	4	0	0	
33	2.242	4	0	0	
48	1.707	4	0	0	
54	1.01	4	0	0	
72	0.775	4	0	0	
80	0.679	4	0	0	
0		5			40
0		5	1	0	
0.25	2.023	5	0	0	
0.5	2.734	5	0	0	
0.75	3.696	5	0	0	
1	3.788	5	0	0	
2	5.87	5	0	0	
3	5.914	5	0	0	
4	4.953	5	0	0	
6	4.68	5	0	0	
8	4.013	5	0	0	
10	3.767	5	0	0	
12	3.092	5	0	0	
24	1.94	5	0	0	
29	1.919	5	0	0	
33	1.841	5	0	0	
48	1.415	5	0	0	
54	0.949	5	0	0	
72	0.587	5	0	0	
80	0.456	5	0	0	
0		6			40
0		6	1	0	
0.25	2.534	6	0	0	
0.5	3.783	6	0	0	

0.75	4.481	6	0	0	
1	4.545	6	0	0	
2	5.431	6	0	0	
3	6.51	6	0	0	
4	6.019	6	0	0	
6	5.55	6	0	0	
8	5.223	6	0	0	
10	4.694	6	0	0	
12	4.492	6	0	0	
24	2.558	6	0	0	
29	2.264	6	0	0	
33	2.04	6	0	0	
48	1.735	6	0	0	
54	0.966	6	0	0	
72	0.489	6	0	0	
80		6	0	1	
0		7			40
0		7	1	0	
0.25	3.354	7	0	0	
0.5	3.9	7	0	0	
0.75	4.547	7	0	0	
1	4.599	7	0	0	
2	5.881	7	0	0	
3	6.245	7	0	0	
4	5.518	7	0	0	
6	4.941	7	0	0	
8	4.574	7	0	0	
10	4.239	7	0	0	
12	3.659	7	0	0	
24	2.136	7	0	0	
29	1.929	7	0	0	
33	1.882	7	0	0	
48	1.202	7	0	0	
54	0.972	7	0	0	
72	0.705	7	0	0	
80	0.658	7	0	0	
0		8			40
0		8	1	0	
0.25	2.385	8	0	0	
0.5	3.262	8	0	0	
0.75	4.094	8	0	0	
1	4.338	8	0	0	
2	5.143	8	0	0	
3	5.377	8	0	0	
4	5.497	8	0	0	
6	5.168	8	0	0	
8	4.719	8	0	0	
10	4.325	8	0	0	
12	4.194	8	0	0	
24	2.487	8	0	0	
29	2.251	8	0	0	
33	2.092	8	0	0	
48	1.475	8	0	0	

54	0.898	8	0	0	
72	0.748	8	0	0	
80	0.645	8	0	0	
0		9			40
0		9	1	0	
0.25	1.909	9	0	0	
0.5	2.167	9	0	0	
0.75	2.384	9	0	0	
1	2.504	9	0	0	
2	4.011	9	0	0	
3	4.262	9	0	0	
4	3.885	9	0	0	
6	3.404	9	0	0	
8	2.812	9	0	0	
10	2.72	9	0	0	
12	2.51	9	0	0	
24	1.877	9	0	0	
29	1.641	9	0	0	
33	1.361	9	0	0	
48	1.147	9	0	0	
54	0.756	9	0	0	
72	0.672	9	0	0	
80	0.552	9	0	0	
0		10			40
0		10	1	0	
0.25	5.095	10	0	0	
0.5	5.925	10	0	0	
0.75	8.58	10	0	0	
1	8.652	10	0	0	
2	9.786	10	0	0	
3	8.606	10	0	0	
4	7.642	10	0	0	
6	6.248	10	0	0	
8	4.99	10	0	0	
10	3.146	10	0	0	
12	2.63	10	0	0	
24	2.083	10	0	0	
29	1.828	10	0	0	
33	1.736	10	0	0	
48	1.118	10	0	0	
54	0.855	10	0	0	
72	0.625	10	0	0	
80	0.356	10	0	0	
0		11			40
0		11	1	0	
0.25	1.19	11	0	0	
0.5	1.77	11	0	0	
1	2.62	11	0	0	
2	4.08	11	0	0	
3	4.46	11	0	0	
4	4.49	11	0	0	
5	3.88	11	0	0	
6	4.45	11	0	0	

7	4.05	11	0	0	
8	3.86	11	0	0	
9	3.75	11	0	0	
10	3.76	11	0	0	
12	3.45	11	0	0	
24	2.27	11	0	0	
29	2.14	11	0	0	
34	1.97	11	0	0	
48	1.26	11	0	0	
72	0.714	11	0	0	
96	0.455	11	0	0	
120	0.328	11	0	0	
144	0.224	11	0	0	
168	0.182	11	0	0	
192		11	1	0	
216	0.183	11	0	0	
0		12			40
0		12	1	0	
0.25	1.92	12	0	0	
0.5	3.14	12	0	0	
1	4.15	12	0	0	
2	4.75	12	0	0	
3	5.41	12	0	0	
4	5.28	12	0	0	
5	6.03	12	0	0	
6	5.71	12	0	0	
7	6.17	12	0	0	
8	6.42	12	0	0	
9	5.98	12	0	0	
10	6.22	12	0	0	
12	5.63	12	0	0	
24	3.18	12	0	0	
29	2.23	12	0	0	
34	1.71	12	0	0	
48	1.08	12	0	0	
72	0.469	12	0	0	
96	0.233	12	0	0	
120	0.11	12	0	0	
144	0.0528	12	0	0	
168		12	1	0	
192		12	1	0	
216		12	1	0	
0		13			40
0		13	1	0	
0.25	1.01	13	0	0	
0.5	1.45	13	0	0	
1	2.28	13	0	0	
2	3.02	13	0	0	
3	3.45	13	0	0	
4	3.46	13	0	0	
5	3.33	13	0	0	
6	3.25	13	0	0	
7	3.18	13	0	0	

8	3.11	13	0	0	
9	3.05	13	0	0	
10	2.99	13	0	0	
12	2.53	13	0	0	
24	1.73	13	0	0	
29	1.43	13	0	0	
34	1.28	13	0	0	
48	0.996	13	0	0	
72	0.71	13	0	0	
96	0.444	13	0	0	
120	0.397	13	0	0	
144	0.356	13	0	0	
168	0.307	13	0	0	
192	0.279	13	0	0	
216	0.263	13	0	0	
0		14			40
0		14	1	0	
0.25	1.1	14	0	0	
0.5	1.84	14	0	0	
1	2.85	14	0	0	
2	3.64	14	0	0	
3	4.17	14	0	0	
4	4.34	14	0	0	
5	4.38	14	0	0	
6	4.22	14	0	0	
7	4.11	14	0	0	
8	4.02	14	0	0	
9	3.85	14	0	0	
10	3.66	14	0	0	
12	3.3	14	0	0	
24	2.19	14	0	0	
29	1.79	14	0	0	
34	1.57	14	0	0	
48	1.02	14	0	0	
72	0.493	14	0	0	
96	0.302	14	0	0	
120	0.232	14	0	0	
144	0.181	14	0	0	
168	0.15	14	0	0	
192	0.139	14	0	0	
216	0.12	14	0	0	
0		15			40
0		15	1	0	
0.25	1.32	15	0	0	
0.5	2.05	15	0	0	
1	3.1	15	0	0	
2	4.32	15	0	0	
3	4.68	15	0	0	
4	4.75	15	0	0	
5	4.76	15	0	0	
6	5.12	15	0	0	
7	4.55	15	0	0	
8	4.75	15	0	0	

9	4.54	15	0	0	
10	4.23	15	0	0	
12	4.17	15	0	0	
24	2.71	15	0	0	
29	2.24	15	0	0	
34	2.01	15	0	0	
48	1.34	15	0	0	
72	0.664	15	0	0	
96	0.387	15	0	0	
120	0.268	15	0	0	
144	0.172	15	0	0	
168	0.116	15	0	0	
192	0.0645	15	0	0	
216		15	1	0	
0		16			40
0		16	1	0	
0.25	0.66	16	0	0	
0.5	1.12	16	0	0	
1	1.67	16	0	0	
2	2.2	16	0	0	
3	2.45	16	0	0	
4	2.58	16	0	0	
5	2.57	16	0	0	
6	2.58	16	0	0	
7	2.58	16	0	0	
8	2.44	16	0	0	
9	2.23	16	0	0	
10	2.31	16	0	0	
12	2.13	16	0	0	
24	1.94	16	0	0	
29	1.63	16	0	0	
34	1.46	16	0	0	
48	1.17	16	0	0	
72	0.687	16	0	0	
96	0.472	16	0	0	
120	0.387	16	0	0	
144	0.277	16	0	0	
168	0.205	16	0	0	
192	0.16	16	0	0	
216	0.123	16	0	0	
0		17			40
0		17	1	0	
0.25	0.883	17	0	0	
0.5	1.45	17	0	0	
1	2.45	17	0	0	
2	3.61	17	0	0	
3	3.82	17	0	0	
4	3.57	17	0	0	
5	3.52	17	0	0	
6	2.59	17	0	0	
7		17	1	1	
8	3.66	17	0	0	
9	3.35	17	0	0	

10	3.54	17	0	0	
12	3.26	17	0	0	
24		17	1	0	
29	2.37	17	0	0	
34	1.89	17	0	0	
48	1.29	17	0	0	
72	0.803	17	0	0	
96	0.455	17	0	0	
120	0.362	17	0	0	
144	0.287	17	0	0	
168	0.23	17	0	0	
192	0.168	17	0	0	
216	0.149	17	0	0	
0		18			40
0		18	1	0	
0.25	1.37	18	0	0	
0.5	1.9	18	0	0	
1	2.8	18	0	0	
2	3.78	18	0	0	
3	4	18	0	0	
4	4.15	18	0	0	
5	3.95	18	0	0	
6	3.91	18	0	0	
7	3.73	18	0	0	
8	3.74	18	0	0	
9	3.57	18	0	0	
10	3.39	18	0	0	
12	3.12	18	0	0	
24	1.97	18	0	0	
29	1.63	18	0	0	
34	1.4	18	0	0	
48	1.07	18	0	0	
72	0.759	18	0	0	
96	0.648	18	0	0	
120	0.52	18	0	0	
144	0.457	18	0	0	
168	0.385	18	0	0	
192	0.295	18	0	0	
216	0.246	18	0	0	
0		19			40
0		19	1	0	
2	3.52	19	0	0	
3	4.6	19	0	0	
4	4.86	19	0	0	
5	4.78	19	0	0	
6	4.34	19	0	0	
9	3.25	19	0	0	
12	3.84	19	0	0	
24	1.95	19	0	0	
30	1.77	19	0	0	
48	1.01	19	0	0	
72	0.692	19	0	0	
96	0.417	19	0	0	

168	0.219	19	0	0	
192	0.167	19	0	0	
0		20			40
0		20	1	0	
2	4.51	20	0	0	
3	5.11	20	0	0	
4	5.21	20	0	0	
5	6	20	0	0	
6	5.04	20	0	0	
9	4.28	20	0	0	
12	4.15	20	0	0	
24	3.04	20	0	0	
30	1.75	20	0	0	
48	0.81	20	0	0	
72	0.572	20	0	0	
96	0.339	20	0	0	
168	0.0927	20	0	0	
192	0.0558	20	0	0	
0		21			40
0		21	1	0	
2	3.32	21	0	0	
3	3.26	21	0	0	
4	3.16	21	0	0	
5	3.66	21	0	0	
6	3.49	21	0	0	
9	3.51	21	0	0	
12	3.5	21	0	0	
24	1.97	21	0	0	
30	1.66	21	0	0	
48	0.935	21	0	0	
72	0.574	21	0	0	
96	0.277	21	0	0	
168	0.0827	21	0	0	
192		21	1	0	
0		22			40
0		22	1	0	
2	3.98	22	0	0	
3	4.07	22	0	0	
4	4.05	22	0	0	
5	4.53	22	0	0	
6	5.18	22	0	0	
9	4.13	22	0	0	
12	5.82	22	0	0	
24	2.06	22	0	0	
30	2.21	22	0	0	
48	1.07	22	0	0	
72	0.536	22	0	0	
96	0.382	22	0	0	
168	0.065	22	0	0	
192	0.0516	22	0	0	
0		23			40
0		23	1	0	
2	2.83	23	0	0	

3	3.81	23	0	0	
4	3.65	23	0	0	
5	3.92	23	0	0	
6	3.64	23	0	0	
9	3.69	23	0	0	
12	3.69	23	0	0	
24	2.06	23	0	0	
30	1.58	23	0	0	
48	1.14	23	0	0	
72	0.796	23	0	0	
96	0.396	23	0	0	
168	0.176	23	0	0	
192	0.17	23	0	0	
0		24			40
0		24	1	0	
2	5.65	24	0	0	
3	6.03	24	0	0	
4	6.33	24	0	0	
5	6.55	24	0	0	
6	6.51	24	0	0	
9	5.9	24	0	0	
12	4.82	24	0	0	
24	2.56	24	0	0	
30	1.67	24	0	0	
48	0.793	24	0	0	
72	0.537	24	0	0	
96	0.323	24	0	0	
168		24	1	0	
192		24	1	0	
0		25			40
0		25	1	0	
2	3.48	25	0	0	
3	3.74	25	0	0	
4	3.93	25	0	0	
5	3.91	25	0	0	
6	4.16	25	0	0	
9	5.14	25	0	0	
12	4.14	25	0	0	
24	2.9	25	0	0	
30	2.75	25	0	0	
48	1.44	25	0	0	
72	0.774	25	0	0	
96	0.355	25	0	0	
168	0.0648	25	0	0	
192		25	1	0	
0		26			40
0		26	1	0	
2	3.49	26	0	0	
3	3.78	26	0	0	
4	3.62	26	0	0	
5	3.67	26	0	0	
6	3.43	26	0	0	
9	3.81	26	0	0	

12	3.24	26	0	0	
24	1.79	26	0	0	
30	1.65	26	0	0	
48	1.21	26	0	0	
72	0.919	26	0	0	
96	0.522	26	0	0	
168	0.0856	26	0	0	
192		26	1	0	
0		27			40
0		27	1	0	
2	4.02	27	0	0	
3	5.04	27	0	0	
4	4.77	27	0	0	
5	4.98	27	0	0	
6	5.46	27	0	0	
9	5.16	27	0	0	
12	4.31	27	0	0	
24	2.55	27	0	0	
30	2.15	27	0	0	
48	0.873	27	0	0	
72	0.46	27	0	0	
96	0.256	27	0	0	
168	0.0648	27	0	0	
192		27	1	0	
0		28			40
0		28	1	0	
2	3.37	28	0	0	
3	4.63	28	0	0	
4	5.39	28	0	0	
5	6.34	28	0	0	
6	5.75	28	0	0	
9	6.34	28	0	0	
12	5.39	28	0	0	
24	3.09	28	0	0	
30	1.89	28	0	0	
48	0.981	28	0	0	
72	0.508	28	0	0	
96	0.308	28	0	0	
168	0.0802	28	0	0	
192		28	1	0	
0		29			40
0		29	1	0	
2	3.68	29	0	0	
3	4.84	29	0	0	
4	4.27	29	0	0	
5	5.19	29	0	0	
6	5.79	29	0	0	
9	5.39	29	0	0	
12	4.63	29	0	0	
24	2.61	29	0	0	
30	2.05	29	0	0	
48	1.22	29	0	0	
72	0.591	29	0	0	

96	0.273	29	0	0	
168		29	1	0	
192		29	1	0	
0		30			40
0		30	1	0	
2	4.69	30	0	0	
3	5.21	30	0	0	
4	5.57	30	0	0	
5	5.41	30	0	0	
6	5.83	30	0	0	
9	5.61	30	0	0	
12	4.98	30	0	0	
24	3.37	30	0	0	
30	2.9	30	0	0	
48	1.27	30	0	0	
72	1.06	30	0	0	
96	0.298	30	0	0	
168	0.0522	30	0	0	
192		30	1	0	
0		31			40
0		31	1	0	
2	3.1	31	0	0	
3	3.51	31	0	0	
4	4	31	0	0	
5	3.77	31	0	0	
6	3.83	31	0	0	
9	4.47	31	0	0	
12	3.11	31	0	0	
24	2.29	31	0	0	
30	2.08	31	0	0	
48	0.597	31	0	0	
72	0.373	31	0	0	
96	0.175	31	0	0	
168	0.0764	31	0	0	
192		31	1	0	
0		32			40
0		32	1	0	
2	3.68	32	0	0	
3	4.18	32	0	0	
4	4.49	32	0	0	
5	3.85	32	0	0	
6	5.08	32	0	0	
9	5.19	32	0	0	
12	4.68	32	0	0	
24	2.69	32	0	0	
30	2.14	32	0	0	
48	1.04	32	0	0	
72	0.448	32	0	0	
96	0.207	32	0	0	
168	0.0504	32	0	0	
192		32	1	0	
0		33			40
0		33	1	0	

2	3.59	33	0	0	
3	4.22	33	0	0	
4	4.83	33	0	0	
5	4.18	33	0	0	
6	4.66	33	0	0	
9	4.25	33	0	0	
12	3.63	33	0	0	
24	2.96	33	0	0	
30	2.13	33	0	0	
48	1.28	33	0	0	
72	0.653	33	0	0	
96	0.36	33	0	0	
168	0.114	33	0	0	
192	0.096	33	0	0	
0		34			40
0		34	1	0	
2	5.44	34	0	0	
3	3.81	34	0	0	
4	2.49	34	0	0	
5	3	34	0	0	
6	2.89	34	0	0	
9	2.85	34	0	0	
12	3.51	34	0	0	
24	1.68	34	0	0	
30	1.28	34	0	0	
48	0.904	34	0	0	
72	0.83	34	0	0	
96	0.546	34	0	0	
168	0.246	34	0	0	
192	0.24	34	0	0	
0		35			40
0		35	1	0	
2	4.24	35	0	0	
3	4.64	35	0	0	
4	5.13	35	0	0	
5	6.22	35	0	0	
6	5.12	35	0	0	
9	5.7	35	0	0	
12	3.86	35	0	0	
24	2.35	35	0	0	
30	1.82	35	0	0	
48	0.936	35	0	0	
72	0.583	35	0	0	
96	0.281	35	0	0	
168	0.1	35	0	0	
192	0.0587	35	0	0	
0		36			40
0		36	1	0	
2	5.66	36	0	0	
3	5.97	36	0	0	
4	6	36	0	0	
5	6.99	36	0	0	
6	6.47	36	0	0	

9	6.78	36	0	0	
12	7.26	36	0	0	
24	3.24	36	0	0	
30	1.13	36	0	0	
48	0.814	36	0	0	
72	0.343	36	0	0	
96	0.203	36	0	0	
168	0.0629	36	0	0	
192		36	1	0	
0		37			40
0		37	1	0	
2	4.21	37	0	0	
3	4.86	37	0	0	
4	5.49	37	0	0	
5	5.37	37	0	0	
6	6.12	37	0	0	
9	6.48	37	0	0	
12	4.7	37	0	0	
24	3.06	37	0	0	
30	1.86	37	0	0	
48	0.87	37	0	0	
72	0.436	37	0	0	
96	0.253	37	0	0	
168	0.0585	37	0	0	
192	0.0838	37	0	0	
0		38			40
0		38	1	0	
2	4.62	38	0	0	
3	4.98	38	0	0	
4	4.55	38	0	0	
5	5.6	38	0	0	
6	5.47	38	0	0	
9	3.86	38	0	0	
12	4.73	38	0	0	
24	2.6	38	0	0	
30	1.65	38	0	0	
48	1.14	38	0	0	
72	0.625	38	0	0	
96	0.3	38	0	0	
168	0.0592	38	0	0	
192	0.0528	38	0	0	
0		39			40
0		39	1	0	
2	3.34	39	0	0	
3	3.37	39	0	0	
4	3.61	39	0	0	
5	3.6	39	0	0	
6	3.52	39	0	0	
9	3.7	39	0	0	
12	3.49	39	0	0	
24	1.89	39	0	0	
30	1.57	39	0	0	
48	0.724	39	0	0	

72	0.524	39	0	0	
96	0.257	39	0	0	
168	0.171	39	0	0	
192	0.073	39	0	0	
0		40			40
0		40	1	0	
2	3.83	40	0	0	
3	3.66	40	0	0	
4	3.81	40	0	0	
5	3.73	40	0	0	
6	3.7	40	0	0	
9	4.51	40	0	0	
12	2.55	40	0	0	
24	1.72	40	0	0	
30	1.41	40	0	0	
48	0.978	40	0	0	
72	0.662	40	0	0	
96	0.455	40	0	0	
168	0.169	40	0	0	
192	0.152	40	0	0	
0		41			40
0		41	1	0	
2	3.82	41	0	0	
3	4.04	41	0	0	
4	3.56	41	0	0	
5	3.87	41	0	0	
6	3.82	41	0	0	
9	4.13	41	0	0	
12	3.72	41	0	0	
24	2.35	41	0	0	
30	2	41	0	0	
48	1.04	41	0	0	
72	0.608	41	0	0	
96	0.0799	41	0	0	
168	0.106	41	0	0	
192	0.337	41	0	0	
0		42			40
0		42	1	0	
2	4.62	42	0	0	
3	4.73	42	0	0	
4	4.62	42	0	0	
5	3.85	42	0	0	
6	4.09	42	0	0	
9	5.19	42	0	0	
12	3.94	42	0	0	
24	2.03	42	0	0	
30	1.57	42	0	0	
48	0.909	42	0	0	
72	0.534	42	0	0	
96	0.201	42	0	0	
168	0.112	42	0	0	
192	0.133	42	0	0	
0		43			40

0		43	1	0	
2	4.21	43	0	0	
3	7.46	43	0	0	
4	6.69	43	0	0	
5	4.92	43	0	0	
6	6.67	43	0	0	
9	4.95	43	0	0	
12	5.89	43	0	0	
24	2.04	43	0	0	
30	2.1	43	0	0	
48	1.1	43	0	0	
72	0.588	43	0	0	
96	0.26	43	0	0	
168	0.0732	43	0	0	
192	0.0632	43	0	0	
0		44			40
0		44	1	0	
2	3.48	44	0	0	
3	3.47	44	0	0	
4	3.91	44	0	0	
5	4.2	44	0	0	
6	4.31	44	0	0	
9	3.7	44	0	0	
12	3.25	44	0	0	
24	1.81	44	0	0	
30	1.44	44	0	0	
48	0.734	44	0	0	
72	0.522	44	0	0	
96	0.341	44	0	0	
168	0.124	44	0	0	
192	0.0938	44	0	0	
0		45			40
0		45	1	0	
2	3.06	45	0	0	
3	4.03	45	0	0	
4	4.04	45	0	0	
5	3.8	45	0	0	
6	4.82	45	0	0	
9	3.27	45	0	0	
12	3.09	45	0	0	
24	1.83	45	0	0	
30	1.38	45	0	0	
48	0.809	45	0	0	
72	0.522	45	0	0	
96	0.338	45	0	0	
168	0.153	45	0	0	
192	0.161	45	0	0	
0		46			40
0		46	1	0	
2	1.53	46	0	0	
3	5.49	46	0	0	
4	4.89	46	0	0	
5	4.63	46	0	0	

6	4.37	46	0	0	
9	3.32	46	0	0	
12	3.14	46	0	0	
24	1.59	46	0	0	
30	1.72	46	0	0	
48	0.845	46	0	0	
72	0.593	46	0	0	
96	0.249	46	0	0	
168	0.166	46	0	0	
192	0.132	46	0	0	
0		47			40
0		47	1	0	
2	6.05	47	0	0	
3	6.45	47	0	0	
4	7.07	47	0	0	
5	9.17	47	0	0	
6	11.2	47	0	0	
9	6.19	47	0	0	
12	4.74	47	0	0	
24	2.27	47	0	0	
30	1.72	47	0	0	
48	1.34	47	0	0	
72	0.853	47	0	0	
96	0.556	47	0	0	
168	0.161	47	0	0	
192		47	1	0	
0		48			40
0		48	0	0	
2	4.34	48	0	0	
3	6.48	48	0	0	
4	6.17	48	0	0	
5	4.94	48	0	0	
6	6.57	48	0	0	
9	5.05	48	0	0	
12	3.57	48	0	0	
24	2.62	48	0	0	
30	2.19	48	0	0	
48	1.24	48	0	0	
72	0.731	48	0	0	
96	0.409	48	0	0	
168	0.0514	48	0	0	
192		48	1	0	
0		49			40
0		49	1	0	
2	6.51	49	0	0	
3	5.12	49	0	0	
4	5	49	0	0	
5	4.94	49	0	0	
6	6.04	49	0	0	
9	4.68	49	0	0	
12	3.71	49	0	0	
24	2.16	49	0	0	
30	2.14	49	0	0	

48	1.07	49	0	0	
72	0.525	49	0	0	
96	0.316	49	0	0	
168	0.219	49	0	0	
192	0.137	49	0	0	
0		50			40
0		50	1	0	
2	2.95	50	0	0	
3	3.82	50	0	0	
4	3.72	50	0	0	
5	4.28	50	0	0	
6	5.38	50	0	0	
9	3.98	50	0	0	
12	3.51	50	0	0	
24	2.11	50	0	0	
30	1.43	50	0	0	
48	0.9	50	0	0	
72	0.715	50	0	0	
96	0.425	50	0	0	
168	0.163	50	0	0	
192	0.15	50	0	0	
